# Probing Electrochemical Potential Differences over the Solid/Liquid Interface in Li-Ion Battery Model Systems

**DOI:** 10.1021/acsami.1c07424

**Published:** 2021-07-12

**Authors:** Ida Källquist, Fredrik Lindgren, Ming-Tao Lee, Andrey Shavorskiy, Kristina Edström, Håkan Rensmo, Leif Nyholm, Julia Maibach, Maria Hahlin

**Affiliations:** † Department of Physics and Astronomy, Uppsala University, 751 20 Uppsala, Sweden; ‡ Department of Chemistry - Ångström, Uppsala University, 751 20 Uppsala, Sweden; § MAX IV Laboratory, Lund University, 225 94 Lund, Sweden; ∥ Institute for Applied Materials (IAM), Karlsruhe Institute of Technology (KIT), Hermann-von-Helmholtz-Platz 1, 76344 Eggenstein-Leopoldshafen, Germany

**Keywords:** electrical double layer, ambient pressure photoelectron spectroscopy, operando spectroscopy, electrochemical potentials, lithium-ion batteries, electrochemical reactions, electrode/electrolyte interface

## Abstract

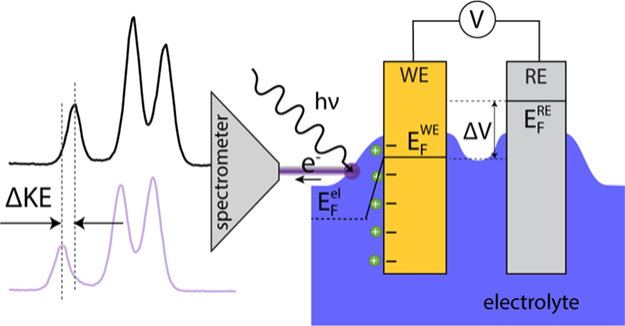

The electrochemical potential difference (Δμ̅) is the driving force for the transfer of a charged species from one phase to another in a redox reaction. In Li-ion batteries (LIBs), Δμ̅ values for both electrons and Li-ions play an important role in the charge-transfer kinetics at the electrode/electrolyte interfaces. Because of the lack of suitable measurement techniques, little is known about how Δμ̅ affects the redox reactions occurring at the solid/liquid interfaces during LIB operation. Herein, we outline the relations between different potentials and show how ambient pressure photoelectron spectroscopy (APPES) can be used to follow changes in Δμ̅_e_ over the solid/liquid interfaces operando by measuring the kinetic energy (KE) shifts of the electrolyte core levels. The KE shift versus applied voltage shows a linear dependence of ∼1 eV/V during charging of the electrical double layer and during solid electrolyte interphase formation. This agrees with the expected results for an ideally polarizable interface. During lithiation, the slope changes drastically. We propose a model to explain this based on charge transfer over the solid/liquid interface.

## Introduction

The driving force for redox reactions in a Li-ion battery (LIB) is the differences in electrochemical potentials between different phases, where a transferable species will strive to move from a phase with higher electrochemical potential to a phase with lower electrochemical potential. The electrochemical potential of a charged species *i* in phase *α* is defined as the energy required to move the species from vacuum at infinity and add it to the phase. The electrochemical potential μ̅_i_^α^ is sometimes separated into one contribution from the chemical potential and one contribution from the electrostatic potential according to the following equation:^1−3^
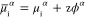
1where μ_i_^α^ is the chemical potential of species *i* in phase α, z is the unit charge, and ϕ^α^ is the electrostatic potential of the phase. To facilitate the comparison to spectroscopy, μ_i_^α^ and μ̅_i_^α^ are given in eV throughout the manuscript. The separation of chemical potential and electrostatic potential is, although only conceptual, useful because a change in chemical potential is local and depends on the change in the chemical environment, while a change in the electrostatic potential of the phase will affect any species with the same charge in the same way.

In LIBs, both non-Faradaic reactions and Faradaic reactions generally occur. The Faradaic reactions in a LIB are the lithiation/delithiation reactions where Li-ions and electrons are transferred between the positive and negative electrodes during charge and discharge. When applying an external voltage between the LIB electrodes, the first process to occur is usually charging of electrical double layers (EDLs) at the electrode/electrolyte interfaces.^4,5^ This process will alter the electrostatic potential difference (Δϕ) between the electrode and electrolyte. When the external voltage is further increased, decomposition of components in the EDL region can occur, and a solid electrolyte interphase (SEI) forms on the surface of the negative electrode as a result of redox reactions.^6−9^ The onset of SEI formation depends on the electrolyte solvents and salts used; however, for typical organic electrolytes used in LIBs, the electrolyte reduction occurs primarily below ∼1 V vs Li^+^/Li.^9,10^ Depending on the reduction potential of the electrode, lithiation can occur before or after SEI formation. If lithiation occurs at a voltage below electrolyte reduction, a well-functioning SEI is essential to obtain a stable and safe battery performance.^10,11^

During the transfer of charged species between the electrode and electrolyte, both μ_i_ and ϕ can be altered. However, ϕ can be changed much faster than μ_i_, and thus, equilibrium can be achieved faster for charged species compared to neutral species.^1,2^ It can be important to note that a species with high mobility (such as an electron in a conductive phase) can be at equilibrium, even though the overall redox reaction is not at equilibrium. The movement of charged species will affect the magnitude and spatial distribution of any potential drop (electrostatic or electrochemical) over an interface and have a fundamental influence on the charge-transfer kinetics for a heterogeneous reaction.^1,2^ Thus, understanding the electrochemical processes occurring at the interfaces is crucial for understanding the properties of any electrochemical device.

Photoelectron spectroscopy (PES) is one of the main tools that have been used to study interfaces in batteries. Early measurements performed already in the 1980s have shown that an EDL that is charged by applying a voltage to an electrode immersed in an electrolyte, in many cases, largely remains at the electrode surface even during measurements performed in ultrahigh vacuum.^12^ The EDL then induces a binding energy (BE) shift of the species outside the outer Helmholtz plane because of the electrochemical potential difference over the EDL.^13,14^ Similarly, PES measurements on cycled electrodes measured post-mortem have shown that Δμ̅ is also present between the electrode and the SEI under high-vacuum conditions, and that the size varies with the electrode state of charge.^15,16^ However, traditional PES does not allow for the presence of volatile compounds such as typical LIB electrolyte solvents. This means that the chemistry of the interface and the interfacial electrostatic properties cannot be studied under real operating conditions with traditional PES. The influence of Δμ̅ on the charge-transfer kinetics and redox reactions occurring at the solid/liquid interfaces in a battery is therefore still far from understood.^17^

Using ambient pressure photoelectron spectroscopy (APPES),^18^ it is possible to include and study liquid LIB electrolytes under realistic conditions. In our previous work, we have used APPES to demonstrate concepts involving the analysis of LIB electrolytes during static conditions,^19,20^ to enable the development of operando APPES studies on LIBs and their electrolytes.^21^ Axnanda et al.,^22^ Lichterman et al.,^23,24^ Favaro et al.,^25^ Ali-Löytty et al.,^26^ and Yu et al.^27^ have made significant contributions to the APPES methodology development for electrochemical interfaces, particularly in studies of changes in the spectroscopic response caused by applying an external voltage. It has been shown that an approximate 1:1 correlation is typically observed between the shifts in the kinetic energy (KE) of the APPES peaks stemming from the electrolyte, and the applied voltage.^23,24,26^

In this work, for the first time, we take an important step toward understanding functional LIB interfaces by analyzing liquid electrolyte-based LIB model systems during lithiation with operando APPES. The change in the KE of the electrolyte peaks is followed as a function of applied voltage to the working electrode (WE). Our results show a deviation from the generally expected shift in the KE of 1 eV/V during lithiation. To explain this, we propose a model based on the change of the electrostatic potential of the electrolyte ϕ^el^ when charge transfer (i.e., Li-ion transfer) occurs as a result of the driving forces to equilibrate the Li-ion electrochemical potential μ̅_Li^+^_.

## Methods

APPES measurements were performed at the HIPPIE beamline at the MAX IV synchrotron facility, Lund, Sweden.^28^ All measurements were performed at a set photon energy of 1800 eV. The beam size was approximately 25 × 50 μm (vertical × horizontal), with an incident angle of 55°, with respect to the sample normal. The photoemission was measured in the normal emission geometry. The spectra were recorded with a Scienta Hipp-3 analyzer, with a cone opening of 0.3 mm. The pressure was maintained as constant as possible at about 0.3 mbar [argon and propylene carbonate (PC) vapor] during all measurements.

The investigated WEs in this work were either a sputter-deposited Au thin film (100 nm) on a Cu substrate or a piece of metallic Cu with a native oxide. The Cu spectra show that the Cu electrode consists of Cu metal as well as Cu oxides and hydroxides, see Figure S1. The counter electrode (CE) was a composite electrode comprising LiNi_1/3_Mn_1/3_Co_1/3_O_2_ (NMC) polyvinylidene difluoride (polymeric binder) and carbon black (conductive additive) coated on an Al substrate. A Li-metal piece was attached to the end of a 1 mm diameter Cu wire and used as a reference electrode (RE). All electrodes were pristine; thus, SEI formation is expected to occur during the first lithiation.

A three-electrode cell setup was used during the operando APPES measurements. The WE was connected to the same electrical ground as the spectrometer. As the electrolyte, a 1 M solution of LiClO_4_ in PC was used. The electrolyte was degassed for several hours in the analysis chamber at a pressure below 1 mbar prior to the measurements. During APPES measurements, the cell was immersed in the electrolyte contained in a polytetrafluoroethylene (PTFE) beaker.

**Figure 1:**
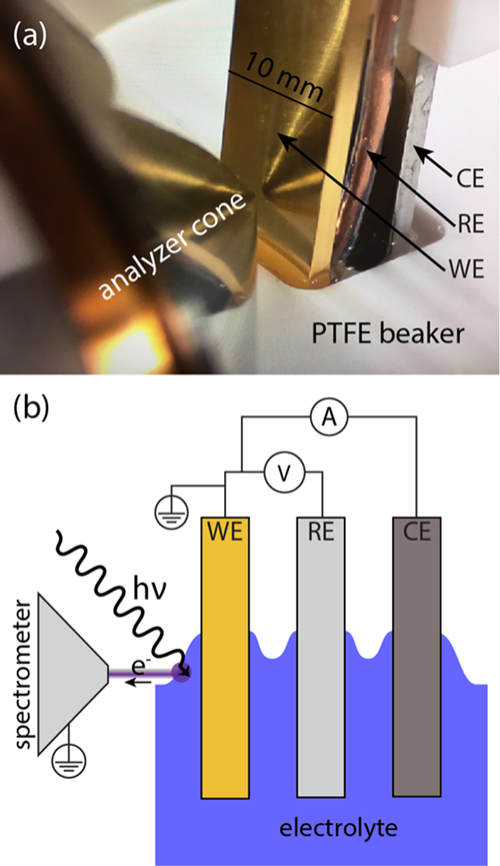
Experimental three-electrode cell setup for operando APPES. (a) Photograph of the operando ambient PES cell setup. (b) An idealized side view of the three-electrode cell setup with the WE, RE, and CE. At the point where the incident synchrotron light (hν) illuminates, the liquid electrolyte meniscus and the outgoing (photo)electrons are indicated.

Figure 1(a) shows a photograph of the three-electrode setup with the Au WE in front of the APPES analyzer front cone. The copper wire onto which the Li-metal RE was attached, and the NMC CE (coated on an Al substrate) are visible behind the WE. All electrodes were attached to the upper sample holder and were dipped into the electrolyte contained in the PTFE beaker (white in picture). In Figure 1(b), the same three-electrode setup is described in a schematic manner (not to scale), where the electrical connections and the angle of the incoming synchrotron light are also illustrated. The APPES measurements were conducted at the meniscus established after a dip-and-pull maneuver,^29^ at a position where the electrolyte meniscus was thick enough to exclude any PE signal from the WE. This means that the liquid layer is thicker than the probing depth; which for electrons with a KE of ∼1500 eV in PC can be estimated as ∼15 nm.^30^ Moreover, for the electrolyte concentrations used here, the EDL thickness is expected to be in in the order of a few Å.^31^ As a result, the components in the EDL do not contribute to the APPES peak intensity or shape.

A BioLogic potentiostat was used to perform the electrochemical measurements. The open-circuit voltage (OCV) between the WE and RE was about 3 V, and the voltage was then decreased in potentiodynamic steps to a series of fixed voltage values. At every fixed voltage, the current was allowed to reach a stable value in the μA range (see Table S1 and Table S2), before APPES measurements were conducted. During electrochemical cycling, the electrodes were dipped into the electrolyte beaker to approximately 10 mm depth. During APPES measurements, the electrodes were partly retracted from the beaker (by approximately 3 mm) to form a liquid meniscus on the WE surface that could be probed with APPES. To make sure the liquid film was continuous during the full operando APPES measurements, the electrodes were redipped to the same position in height every time the voltage was changed. For APPES measurements, the sample (WE) was stepwise moved sideways to ensure that the analyzed spot was fresh and free from radiation damage. On each sample spot, the C 1s, O 1s, and Cl 2p regions were measured. An entire measurement series took approximately 17 h for the Au system and 5 h for the Cu system.

All spectra are presented as measured, that is, no energy calibration is applied. Data analysis was carried out using Igor Pro (version 6.37). To fit the PC molecule emissions in the C 1s spectra, three peaks with a fixed 1:2:1 intensity ratio were used, and their energetic spacing was fixed to the KEs of 3.7 and 5.7 eV higher KE compared to the carbonate peak. Voigt profiles were used for peak fitting with a Lorentzian contribution to the full width at half maximum [FWHM(L)] of 0.1 eV and a Gaussian contribution of FWHM(G) = 1.22(3) eV. Additional components were required to fully account for the measured intensities, and these additional peaks essentially correspond to adventitious carbons on the electrolyte surface. The same FWHM values were applied to the fits of the adventitious carbons. The KE of the fitted carbonate peak in the C 1s spectra was used to present the electrolyte peak positions as a function of the applied voltage. O 1s and Cl 2p spectra were also recorded and behave in a similar fashion as the C 1s spectra. These spectra do not add any new results and are therefore not shown.

## Results

In this study, we investigate the change in electron electrochemical potential difference Δμ̅_e_ over the WE/electrolyte interface as a function of applied voltage Δ*V* to the WE. A liquid meniscus is created by the dip-and-pull method to allow for APPES measurements on the electrolyte (see details in Methods section). APPES measurements are performed on a thick part of the liquid meniscus, where the electrolyte is assumed to exhibit bulk properties (i.e., constant concentration). For these conditions, the measured shifts in KE will correspond to the change in Δμ̅_e_ between the bulk electrode and bulk electrolyte. However, it can be noted that after SEI formation and/or lithiation, more than two phases are present in the interfacial region, and the measured Δμ̅_e_ will then be the sum of several individual contributions stemming from the different phase boundaries (e.g., WE/SEI and SEI/electrolyte).

To analyze the changes in Δμ̅_e_ as a function of the applied voltage and to elucidate the influence of SEI formation on Δμ̅_e_ between the WE and bulk electrolyte, two LIB model systems are studied with operando APPES. The model systems constitute of a three-electrode setup, including a metallic WE, a Li^+^/Li RE, and an NMC CE. In this work, all voltages are given versus the Li^+^/Li RE.

In the first model system, a gold thin film is used as the WE to study the formation of a Li*_x_*Au alloy (i.e., Au + *x* Li^+^ + *x* e^–^ ↔ Li*_x_*Au). Lithiation occurs around and below 0.2 V,^32^ coinciding with typical operating voltages for negative electrodes in LIBs. Because of the low lithiation potential, SEI formation will occur before the alloying reaction between Li and Au. For the electrolyte used in this work (1 M LiClO_4_ in PC), the onset of electrolyte reduction has been observed between 1.6 and 1.0 V,^33,34^ depending on trace amounts of water. In our model systems, the major SEI formation is assumed to occur around 0.6 V, based on the shapes of the cyclic voltammograms (see Figure S2 and S3).

In the second model system, a metallic copper electrode with a native copper oxide acts as the electroactive material. When the applied voltage is lowered from OCV, copper oxide reduction (or conversion) reactions (e.g., CuO + 2 Li^+^ + 2 e^–^ ↔ Cu + Li_2_O or Cu_2_O + 2 Li^+^ + 2 e^–^ ↔ 2 Cu + Li_2_O) will occur first above 1.4 V, followed by electrolyte reduction and SEI formation around 0.6 V (see Figure S2 and S3).

**Figure 2:**
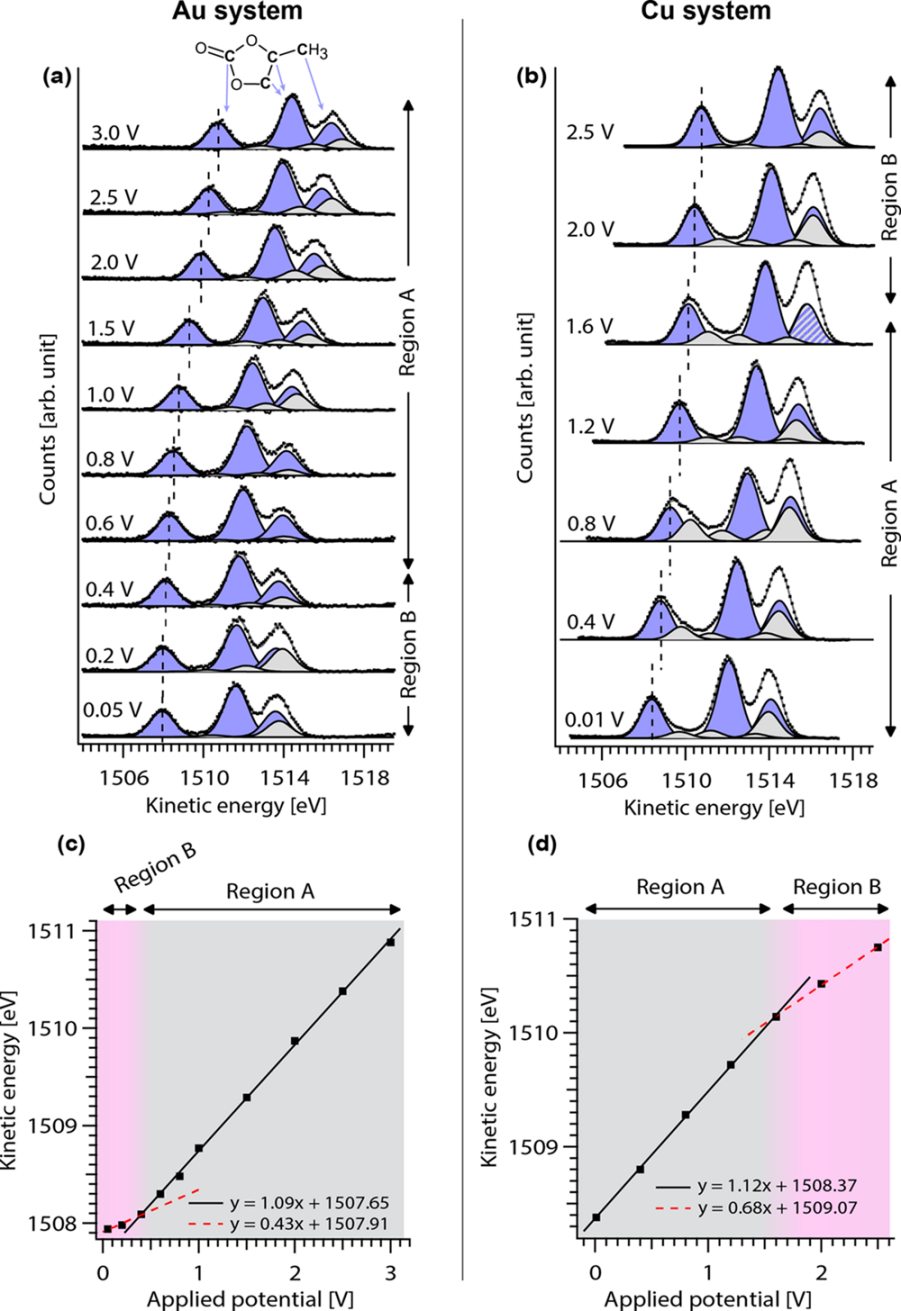
Operando APPES results for the electrolyte in the Au (left) and Cu (right) setup. C 1s spectra measured on the electrolyte for the Au setup (a) and Cu setup (b). At 1.6 V, the striped peak (∼1516 eV) indicates that the PC hydrocarbon peak and the adventitious hydrocarbon peaks overlap. The PC molecule with arrows indicate the corresponding fitted peaks. The KE of the fitted carbonate peaks in the C 1 s spectra [indicated by dashed lines in (a) and (b)] shown as a function of the applied voltage for the Au setup (c) and Cu setup (d).

The APPES results are presented in Figure 2. Figure 2(a,b) depict fitted electrolyte C 1s spectra for the Au WE and Cu WE systems, respectively. The components originating from the PC molecule (top in Figure 2(a)) are presented as purple peaks with an intensity ratio of 1:2:1,^20^ whereas the gray fitted peaks represent adventitious carbons (essentially hydrocarbons) deposited on the electrolyte surface.^35^ The peak positions shown in Figure 2(a,b) shift to lower KE with decreasing applied voltage. It can be noted that the adventitious carbon peaks essentially shift together with the PC peaks for all voltages, and thus, they should be located outside the charged interface layer at the WE/electrolyte interface.

In Figure 2(c,d), the KE of the fitted carbonate peak (from the PC molecule) is plotted as a function of the applied voltage for the two systems. Here, we highlight two observations: first, for both the Au and the Cu WEs, two regions with different dependencies between the change in the KE and applied voltage appear, denoted as Region A and Region B. Second, the dependency in Region A is clearly linear and similar for both systems (close to 1 eV/V), while the dependencies in Region B are different and deviate significantly from a 1 eV/V slope, as indicated in the figures using linear approximations (about 0.4 and 0.7 eV/V for Au and Cu, respectively).

Considering the Au case, lithiation is known to occur at 0.2 V and below.^32^ This is seen in our measurements as a considerable increase in current (see Figure S4 in the SI), as well as a color change of the electrode from gold to gray.^36^ The latter confirms that the electrochemical cell in this experiment functions as expected. APPES measurements are thus performed operando during Au lithiation, although after the current has decayed to limiting current conditions. At the voltages where lithiation occurs (i.e., data points 0.2 and 0.05 V), we see a deviation from the ∼1 eV/V dependence (Figure 2(c)). In the same potential region for the Cu WE system (Figure 2(d)), ∼1 eV/V is followed. In the copper case, a slope lower than 1 eV/V is seen in the potential region between 1.6 V and 2.5 V. This is the potential region where the native copper oxides are known to be subject to conversion reactions.^37,38^ Because the copper oxide layer is very thin, the lithiation current will be lower compared to the gold case. The conversion reaction of the thin layer of Cu oxide to Li oxide can then be completed during the 1.6 V potential step. After lithiation, further lowering the voltage will result in electrostatic charging of a double layer at the electrode surface. In this potential region below 1.6 V, a constant slope of approximately 1 eV/V is seen.

## Discussion

This study combines spectroscopy with electrochemistry; thus, for readers of both fields to understand the results, it is important that a common language is established, and that it is clearly and carefully specified which potential is referred to.^3^ To facilitate this, we will begin this discussion by presenting some important background and definitions that are necessary to correlate the electrochemical measurements to the APPES measurements.

In electrochemistry, the electrochemical potential difference of electrons Δμ̅_e_ in different electrodes is measured/controlled by the voltage. Because the voltage difference Δ*V* corresponds to the work required to move a unit positive charge from one electrode to the other, the negative of the voltage is equal to the electron electrochemical potential difference Δμ̅_e_ between the electrodes.^3,39^ The voltage is usually measured versus a RE. The RE is chosen as an electrode that behaves like an ideally nonpolarizable electrode, that is, μ̅_e_ is (essentially) constant. In this way, when the voltage of the electrode under study (WE) is measured versus that of the RE, any change in voltage between them will stem from a change in μ̅_e_ of the WE.

With APPES, different electron BEs are measured with respect to the Fermi level, *E*_F_, of the spectrometer. To facilitate the interpretation of spectra, the measured sample is usually electrically connected to the spectrometer, so that *E*_F_ of the spectrometer is aligned with *E*_F_ of all electrically conductive phases of the sample. In this case, the BE of an electron, defined as the energy difference between the core level and Fermi level of the phase, can be measured directly. However, if the sample contains any phases that do not conduct electrons, these may have a different E_F_ as a result of an electrochemical potential difference over the phase boundary. In this case, an (internal) energy calibration may be necessary to better interpret the BEs.^16^

When spectroscopy is combined with electrochemistry, the results can be interpreted by considering the electrochemical potential of the electron, μ̅_e_. By definition, μ̅_e_ of a phase is equal to the Fermi level, *E*_F_, of the phase.^1−3^ Furthermore, the splitting of the electrochemical potential into a chemical and an electrostatic potential (see eq 1) can help the interpretation of the spectroscopic measurements. Changes in ϕ are a result of the addition/removal/redistribution of charges (e.g., as a result of applying a bias or building up an EDL). If the voltage of a phase is changed by a purely electrostatic contribution Δϕ, the KE of all photoelectrons stemming from this phase will shift by exactly the same amount (1 eV/V). Changes in μ_e_ instead stem from a change in the local chemical composition of the phase and occur during Faradaic reactions. A change in μ_e_ can be identified by the chemical shifts that can be seen in (AP)PES when the chemical environment of a species is changed. However, in this case, the changes in BE for different core levels can vary, depending on how they are affected by the chemical reaction. Any shift in BE can be related to the measured shifts in KE according to ΔBE = −ΔKE.

From these definitions, we can conclude that if we want to measure the true electrochemical potential difference between two phases with APPES, the Fermi levels should be probed. However, useful information can still be gained by measuring a core level, because these will shift equally with μ̅_e_ = *E*_F_ if only ϕ is changed. These conditions are achieved if the measured phase has a constant chemical potential (i.e., constant pressure, temperature, and chemical composition).

To interpret our operando APPES measurements based on these definitions, it is first necessary to make some assumptions for our electrochemical setup. In our study, we can assume that μ̅_e_ is constant within both WEs (Au and Cu) because conductive metals are used. Furthermore, μ_e_ is also constant for pure bulk metals^1^ (electron concentration does not change appreciably) and a change in voltage will thereby only affect ϕ when non-Faradaic reactions occur. In addition, we assume that the RE behaves as an ideally nonpolarizable electrode.^1^ This assumption can be made because we have a pure Li metal (reduced form, Li^0^) in contact with a high concentration of Li^+^ (oxidized form) that serves as a buffer for the negligible current that goes through the RE during measurements. In this way, μ̅_e_^RE^ (versus an arbitrary reference) remains constant.

The electrolyte is an electronic insulator and does not contain any free electrons. However, electrons can still be added to the electrolyte if the voltage is lowered below its reduction potential, and thus, one can still refer to a chemical potential of the electron in the electrolyte, μ_e_^el^. If the electrolyte composition is constant, μ_e_^el^ will remain constant (temperature and pressure are constant during the measurements). For our systems, Li-ion transport is faster in the bulk electrolyte than in the WEs, and thus, Li^+^ diffusion in the WE will be the rate-determining step of the redox reaction. Because we perform APPES measurements when the current has decayed to a stable value (i.e., limiting current conditions), we can assume that the electrolyte composition is constant; hence, μ_e_^el^ is also constant. In this case, any shift in μ̅_e_^el^ will be caused by a shift in ϕ^el^. This means that we can measure any core level of the electrolyte to evaluate Δμ̅_e_ between the WE and electrolyte as a function of applied voltage Δ*V*.

**Figure 3:**
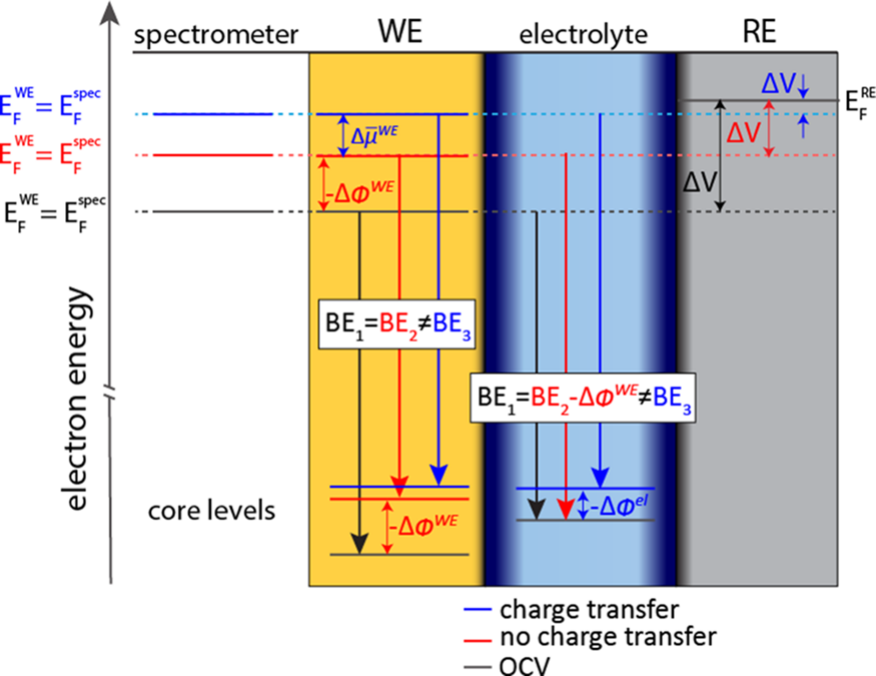
Schematic illustration of relevant energy levels and their measured shifts in BE (ΔBE = −ΔKE) during electrochemical cycling. Red lines and arrows indicate the shifts when no charge transfer occurs. In this case, only μ̅_e_^WE^ is affected by the change in the applied voltage (Δ*V*). Because no redox reactions occur, all chemical potentials are constant and Δμ̅_e_^WE^ = – Δϕ^WE^. Blue lines and arrows indicate the shifts during charge transfer (lithiation). In this case, both μ_e_ and ϕ can be changed for both phases. For the WE, μ_e_^WE^ is changed as the chemical composition changes during lithiation. In this case, the core levels of the WE will, in general, not change equally to the Fermi level. Depending on the magnitude of Δμ_e_^WE^, relative to ΔV, ϕ^WE^ may also change to account for the total change in the applied voltage. For the electrolyte, the chemical composition is constant, and thus, Δμ̅_e_^el^ = – Δϕ^el^, and all electron energy levels of the electrolyte shift equally.

In Region A in our measurements, only EDL charging occurs. In this voltage region, Δ*V* will be changed by changing ϕ^WE^ by the addition/removal of charges to the WE surface. Without charge transfer, μ̅_e_^el^ relative to the RE remains unaffected. Thus, when no charge transfer occurs, the only electron energy levels that are affected by the change in voltage are those in the WE. This case is illustrated in red in Figure 3. However, because μ̅_e_^WE^ = *E*_F_^WE^ serves as a reference point for all APPES measurements, the change in μ̅_e_^WE^ will cause a shift in the measured KE of the bulk electrolyte PE peaks. Thus, if μ̅_e_^el^ remains constant, a relationship of exactly 1 eV/V between the measured ΔKE of the electrolyte PE peaks and ΔV is expected. Previous studies investigating electrochemical systems with operando APPES have also reported essentially an ∼1 eV/V relationship between changes in voltage and changes in KE of the electrolyte peaks.^23,24,26^ The results presented in Figure 2(c,d) show that the obtained slopes of the fitted straight lines are slightly larger than the expected value of 1 eV/V. The origin of the additional shift in KE is unclear and requires further investigation. However, because the EDL theoretically should not be charged to a larger value than the applied external voltage, we believe that this deviation stems from the measurement error of our setup.

The approximate 1 eV/V slope in Region A is followed for both systems also at cell voltages where SEI formation is expected to be the most intense (around 0.6 V). This implies that the SEI formation does not significantly influence the relationship between Δμ̅_e_ and the applied voltage over the full WE/electrolyte interface region. This can be expected as the SEI is meant to work as an insulating passivation layer, preventing the transport of electrons between the WE and the bulk electrolyte. In this way, further charge transfer of electrons to the electrolyte can be limited after SEI formation, and the electrochemical potential of the bulk electrolyte remains unaffected. In this case, a 1 eV/V slope is expected, just as in the case of only EDL charging. However, it is important to note that in this work, only the total Δμ̅_e_ between the WE and electrolyte is probed, not the interface directly. If we were able to probe the WE/SEI/electrolyte interface directly, it would also be possible to evaluate the spatial distribution of the potential drop over the charged interface layer. This was not possible for our experimental setup; however, previous studies performed ex situ suggest that a large part of the EDL is located at the WE/SEI interface.^15,16^ Furthermore, if the spectral contributions from the SEI could be separated from the contributions from the electrolyte, it would also be possible to estimate Δμ̅_e_between SEI/electrolyte, which could be of interest to understand in detail how the SEI functions in an LIB.

In Region B, which is highlighted in pink in Figure 2(c,d), the fitted linear slopes are considerably smaller than 1 eV/V for both the Au and Cu systems. In both cases, Region B coincides with the potential region where lithiation occurs, indicating that Δμ̅_e_ follows a different behavior when charge transfer occurs at the WE/electrolyte interface. Because Region B is located at different potential regions depending on the WE used, we can exclude that the deviation from a 1 eV/V slope is an effect stemming from the electrolyte’s behavior at specific potentials.

In other studies, deviations from a 1 eV/V slope have been explained as a result of an iR_s_ drop, affecting the voltage measured between the WE and RE.^26,40^ This can be the case for APPES measurements that are performed during high current densities at a measurement spot located far away from the bulk electrolyte surface. Furthermore, limited mass transport in the thin liquid meniscus leads to high overpotentials and limited current densities and can also cause ion depletion over time.^29,41,42^ To avoid these effects, we use a slightly different measurement approach (see the Methods section). During our measurements, the WE is redipped between every potential step, and the current is allowed to decay while the electrodes are immersed in the electrolyte beaker. In addition, APPES measurements are performed as close to the bulk electrolyte surface as possible (3–4 mm above the electrolyte surface, limited by the measurement setup) on a thick part of the electrolyte meniscus. With a maximum current density of 0.01 mA/cm^2^ and a high electrolyte concentration of 1 M, we are confident that the iR_s_ drop in the bulk solution can be neglected even in the liquid meniscus, which is in line with the study performed by Ali-Löytty et al.^26^ Especially, in the Cu case, it is clear that there has to be another mechanism responsible for the behavior during lithiation because the current during Cu conversion (Region B) is lower compared to the currents during measurements in Region A (see Table S2). Thus, we rule out iR_s_ drop as an explanation for the deviation from the 1 eV/V slope in Region B (see discussion in the Supporting Information).

We believe that the deviation from the 1 eV/V slope during lithiation reactions stems from the charge transfer occurring at the WE/electrolyte interface, leading to a change in the electrostatic potential of the electrolyte ϕ^el^. Here, we present a possible model to explain this, based on the equilibration of μ̅_Li+_ between the WE and electrolyte.

In an LIB, Li ions are the transferable species at the electrode/electrolyte interfaces. If a sufficiently low voltage is applied to the negative electrode, μ̅_Li+_^WE^ will be lowered below μ̅_Li+_^el^, and there will be a driving force moving Li ions from the electrolyte to the WE. During lithiation, both μ_e_^WE^ and ϕ^WE^ can be changed as Li ions, and electrons are added to the WE. Because of the charge transfer of Li ions from the electrolyte to the electrode, ϕ^el^ can also be changed as a result of the strive to equilibrate the electrochemical potentials of Li ions at the WE/electrolyte interface. These shifts are illustrated in blue in Figure 3. If ϕ^el^ of the electrolyte is changed, both μ̅_Li+_^el^ and μ̅_e_^el^ will be changed. Given that the chemical composition of the electrolyte is constant, μ_e_^el^ is constant, and it follows that Δμ̅_e_^el^ = – Δϕ^el^. The magnitude of Δμ̅_e_^el^ can then be determined from operando APPES according to the following equations:
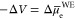
2
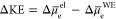
3
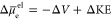
4where eq 2 is measured using a potentiostat, eq 3 is measured by APPES, and eq 4 is given from the combination of these two measurements/equations. Thus, from our results, we can identify that we have a shift in μ̅_e_^el^ (vs Li^+^/Li) during charge transfer for both the Cu and Au systems, where a lower slope in KE vs applied voltage corresponds to a larger change of μ̅_e_^el^. Because the driving force for the change in μ̅_e_^el^ is suggested to stem from differences in μ̅_Li+_ between the WE and electrolyte, the deviation from a 1 eV/V slope could also be used to assess changes in μ̅_Li+_^WE^ during lithiation as a function of the voltage. This could be used as a tool to further understand the lithiation mechanism of the electroactive materials and will be the subject of future studies.

The detected change in μ̅_e_^el^ (vs the RE) is somewhat surprising because it is often assumed that the potential drop at the RE/electrolyte interface remains constant. However, this is only true if these phases are in equilibrium. This would be the case if electrons could flow in/out of the RE to establish the thermodynamic equilibrium of the Li ↔ Li^+^ + e^–^ reaction. However, because of the limited current through the RE, equilibrium may not be achieved. During lithiation, μ̅_e_^el^ would instead be connected to μ̅_e_^WE^ via the redox reactions occurring at the WE/electrolyte interface. Finally, it should be noted that this model builds on the previously made assumptions that (i) Δμ̅_e_^RE^ = 0, (ii) iR_s_ drop in solution can be neglected, and (iii) the chemical composition of the electrolyte measured with APPES is constant and is only valid under these conditions.

## Conclusions

This work demonstrates that fundamental properties such as Δμ̅_e_ over the solid/liquid interface can be probed by operando APPES, even without direct access to the interface itself. We have presented a methodology for using operando APPES as a tool to probe charge transfer over the WE/electrolyte interface during lithiation in two LIB model systems. The results show that when charge transfer occurs, a change in the applied voltage to the WE can also cause a change in the electrochemical potential of the electrolyte. This is reflected in the measured shifts of the electrolyte peak positions that then deviates from the 1 eV/V slope expected for an ideal polarizable interface. Based on our suggested model to explain this, the change in μ̅_e_^el^ can be directly evaluated from the operando APPES measurements for certain measurement conditions. To develop our suggested model, additional measurements will be performed where also the dependence of current and setup limitations are investigated.

As the potential differences over the interface will govern the charge transfer properties, we believe that the developed methodology will provide new means to further understand the mechanisms behind EDL charging, SEI formation, and lithiation in LIBs. To fully take advantage of the possibilities with operando APPES, it would be desirable to probe the solid/liquid interface directly. In this way, it would be possible to identify, for example, if EDL charging occurs or if μ_e_ of the WE changes, and couple this information with the changes in Δμ̅_e_ during operando measurements. We propose this as an essential future direction to develop more detailed models of the interfacial chemistry in LIB systems.

All relevant data are available from the authors upon request.

## Supporting Information Available

Characterization of the Cu WE; results from electrochemical measurements; and discussion of resistances in the electrochemical cell (PDF)

## References

[ref1] Bard A. J.; Faulkner L. R. Electrochemical Methods: Fundamentals and Applications; 2nd ed.; Wiley: New York, 2001.

[ref2] Koryta J.; Dvořák J.; Kavan L. Principles of Electrochemistry; John Wiley & Sons Inc, 1993.

[ref3] Boettcher S. W.; Oener S. Z.; Lonergan M. C.; Surendranath Y.; Ardo S.; Brozek C.; Kempler P. A. Potentially Confusing: Potentials in Electrochemistry. ACS Energy Lett. 2021, 6, 261–266. 10.1021/acsenergylett.0c02443.

[ref4] Quiroga M. A.; Xue K.-H.; Nguyen T.-K.; Tułodziecki M.; Huang H.; Franco A. A. A Multiscale Model of Electrochemical Double Layers in Energy Conversion and Storage Devices. J. Electrochem. Soc. 2014, 161, E3302–E3310. 10.1149/2.029408jes.

[ref5] Marcicki J.; Conlisk A. T.; Rizzoni G. A Lithium-Ion Battery Model Including Electrical Double Layer Effects. J. Power Sources 2014, 251, 157–169. 10.1016/j.jpowsour.2013.11.001.

[ref6] Peled E. The Electrochemical Behavior of Alkali and Alkaline Earth Metals in Nonaqueous Battery Systems—the Solid Electrolyte Interphase Model. J. Electrochem. Soc. 1979, 126, 2047 10.1149/1.2128859.

[ref7] Peled E. Advanced Model for Solid Electrolyte Interphase Electrodes in Liquid and Polymer Electrolytes. J. Electrochem. Soc. 1997, 144, L208 10.1149/1.1837858.

[ref8] Martin W. The Solid Electrolyte Interphase – the Most Important and the Least Understood Solid Electrolyte in Rechargeable Li Batteries. Z. Phys. Chem. 2009, 223, 1395–1406.

[ref9] Verma P.; Maire P.; Novak P. A Review of the Features and Analyses of the Solid Electrolyte Interphase in Li-Ion Batteries. Electrochim. Acta 2010, 55, 6332–6341. 10.1016/j.electacta.2010.05.072.

[ref10] Gauthier M.; Carney T. J.; Grimaud A.; Giordano L.; Pour N.; Chang H.-H.; Fenning D. P.; Lux S. F.; Paschos O.; Bauer C.; Maglia F.; Lupart S.; Lamp P.; Shao-Horn Y. Electrode–Electrolyte Interface in Li-Ion Batteries: Current Understanding and New Insights. J. Phys. Chem. Lett. 2015, 6, 4653–4672. 10.1021/acs.jpclett.5b01727. 26510477

[ref11] Goodenough J. B.; Kim Y. Challenges for Rechargeable Li Batteries. Chem. Mater. 2010, 22, 587–603. 10.1021/cm901452z.

[ref12] Hansen W. N. The Emersed Double Layer. J. Electroanal. Chem. Interf. Electrochem. 1983, 150, 133–140. 10.1016/S0022-0728(83)80197-1.

[ref13] D’Agostino A. T.; Hansen W. N. Observation of Systematic Electrochemically Induced Binding Energy Shift in the Xps Spectra of Emersed Cs+ Double Layer Species. Surf. Sci. 1986, 165, 268–276. 10.1016/0039-6028(86)90674-6.

[ref14] Zhou W.; Kolb D. M. Influence of an Electrostatic Potential at the Metal/Electrolyte Interface on the Electron Binding Energy of Adsorbates as Probed by X-Ray Photoelectron Spectroscopy. Surf. Sci. 2004, 573, 176–182. 10.1016/j.susc.2004.09.022.

[ref15] Maibach J.; Lindgren F.; Eriksson H.; Edstrom K.; Hahlin M. Electric Potential Gradient at the Buried Interface between Lithium-Ion Battery Electrodes and the Sei Observed Using Photoelectron Spectroscopy. J. Phys. Chem. Lett. 2016, 7, 1775–1780. 10.1021/acs.jpclett.6b00391. 27104985

[ref16] Lindgren F.; Rehnlund D.; Källquist I.; Nyholm L.; Edström K.; Hahlin M.; Maibach J. Breaking Down a Complex System: Interpreting Pes Peak Positions for Cycled Li-Ion Battery Electrodes. J. Phys. Chem. C 2017, 121, 27303–27312. 10.1021/acs.jpcc.7b08923.

[ref17] Gauthier M.; Carney T. J.; Grimaud A.; Giordano L.; Pour N.; Chang H.-H.; Fenning D. P.; Lux S. F.; Paschos O.; Bauer C.; Maglia F.; Lupart S.; Lamp P.; Shao-Horn Y. Electrode–Electrolyte Interface in Li-Ion Batteries: Current Understanding and New Insights. J. Phys. Chem. Lett. 2015, 6, 4653–4672. 10.1021/acs.jpclett.5b01727. 26510477

[ref18] Salmeron M.; Schlögl R. Ambient Pressure Photoelectron Spectroscopy: A New Tool for Surface Science and Nanotechnology. Surf. Sci. Rep. 2008, 63, 169–199. 10.1016/j.surfrep.2008.01.001.

[ref19] Maibach J.; Xu C.; Eriksson S. K.; Ahlund J.; Gustafsson T.; Siegbahn H.; Rensmo H.; Edstrom K.; Hahlin M. A High Pressure X-Ray Photoelectron Spectroscopy Experimental Method for Characterization of Solid-Liquid Interfaces Demonstrated with a Li-Ion Battery System. Rev. Sci. Instrum. 2015, 86, 044101 10.1063/1.4916209. 25933870

[ref20] Maibach J.; Kallquist I.; Andersson M.; Urpelainen S.; Edstrom K.; Rensmo H.; Siegbahn H.; Hahlin M. Probing a Battery Electrolyte Drop with Ambient Pressure Photoelectron Spectroscopy. Nat. Commun. 2019, 10, 10803 10.1038/s41467-019-10803-y. PMC6626006 31300638

[ref21] Dietrich P. M.; Gehrlein L.; Maibach J.; Thissen A. Probing Lithium-Ion Battery Electrolytes with Laboratory near-Ambient Pressure Xps. Crystals 2020, 10, 1056 10.3390/cryst10111056.

[ref22] Axnanda S.; Crumlin E. J.; Mao B. H.; Rani S.; Chang R.; Karlsson P. G.; Edwards M. O. M.; Lundqvist M.; Moberg R.; Ross P.; Hussain Z.; Liu Z. Using ″Tender″ X-Ray Ambient Pressure X-Ray Photoelectron Spectroscopy as a Direct Probe of Solid-Liquid Interface. Sci. Rep. 2015, 5, 9788 10.1038/srep09788. 25950241 PMC4650780

[ref23] Lichterman M. F.; Hu S.; Richter M. H.; Crumlin E. J.; Axnanda S.; Favaro M.; Drisdell W.; Hussain Z.; Mayer T.; Brunschwig B. S.; Lewis N. S.; Liu Z.; Lewerenz H. J. Direct Observation of the Energetics at a Semiconductor/Liquid Junction by Operando X-Ray Photoelectron Spectroscopy. Energy Environ. Sci. 2015, 8, 2409–2416. 10.1039/C5EE01014D.

[ref24] Lichterman M. F.; Richter M. H.; Brunschwig B. S.; Lewis N. S.; Lewerenz H.-J. Operando X-Ray Photoelectron Spectroscopic Investigations of the Electrochemical Double Layer at Ir/Koh(Aq) Interfaces. J. Electron Spectrosc. Relat. Phenom. 2017, 221, 99–105. 10.1016/j.elspec.2017.03.011.

[ref25] Favaro M.; Jeong B.; Ross P. N.; Yano J.; Hussain Z.; Liu Z.; Crumlin E. J. Unravelling the Electrochemical Double Layer by Direct Probing of the Solid/Liquid Interface. Nat. Commun. 2016, 7, 12695 10.1038/ncomms12695. 27576762 PMC5013669

[ref26] Ali-Löytty H.; Louie M. W.; Singh M. R.; Li L.; Sanchez Casalongue H. G.; Ogasawara H.; Crumlin E. J.; Liu Z.; Bell A. T.; Nilsson A.; Friebel D. Ambient-Pressure Xps Study of a Ni–Fe Electrocatalyst for the Oxygen Evolution Reaction. J. Phys. Chem. C 2016, 120, 2247–2253. 10.1021/acs.jpcc.5b10931.

[ref27] Yu L.; Takagi Y.; Nakamura T.; Sekizawa O.; Sakata T.; Uruga T.; Tada M.; Iwasawa Y.; Samjeské G.; Yokoyama T. Non-Contact Electric Potential Measurements of Electrode Components in an Operating Polymer Electrolyte Fuel Cell by near Ambient Pressure Xps. Phys. Chem. Chem. Phys. 2017, 19, 30798–30803. 10.1039/C7CP05436J. 29134220

[ref28] Zhu S.; Scardamaglia M.; Kundsen J.; Sankari R.; Tarawneh H.; Temperton R.; Pickworth L.; Cavalca F.; Wang C.; Tissot H.; Weissenrieder J.; Hagman B.; Gustafson J.; Kaya S.; Lindgren F.; Kallquist I.; Maibach J.; Hahlin M.; Boix V.; Gallo T.; Rehman F.; D’Acunto G.; Schnadt J.; Shavorskiy A. Hippie: A New Platform for Ambient-Pressure X-Ray Photoelectron Spectroscopy at the Max Iv Laboratory. J. Synchrotron Radiat. 2021, 28, 624–636. 10.1107/S160057752100103X. 33650575 PMC7941293

[ref29] Favaro M.; Abdi F. F.; Crumlin E. J.; Liu Z.; van de Krol R.; Starr D. E. Interface Science Using Ambient Pressure Hard X-Ray Photoelectron Spectroscopy. Surfaces 2019, 2, 78–99. 10.3390/surfaces2010008.

[ref30] Powell C. J.; Jablonski A. Nist Electron Inelastic-Mean-Free-Path Database 71, Version 1.0; NIST, 1999.

[ref31] Brown M. A.; Goel A.; Abbas Z. Effect of Electrolyte Concentration on the Stern Layer Thickness at a Charged Interface. Angew. Chem., Int. Ed. 2016, 55, 3790–3794. 10.1002/anie.201512025. 26880184

[ref32] Bach P.; Stratmann M.; Valencia-Jaime I.; Romero A. H.; Renner F. U. Lithiation and Delithiation Mechanisms of Gold Thin Film Model Anodes for Lithium Ion Batteries: Electrochemical Characterization. Electrochim. Acta 2015, 164, 81–89. 10.1016/j.electacta.2015.02.184.

[ref33] Aurbach D.; Gottlieb H. The Electrochemical-Behavior of Selected Polar Arotic Systems. Electrochim. Acta 1989, 34, 141–156. 10.1016/0013-4686(89)87079-3.

[ref34] Zhang X.; Kostecki R.; Richardson T. J.; Pugh J. K.; Ross P. N. Electrochemical and Infrared Studies of the Reduction of Organic Carbonates. J. Electrochem. Soc. 2001, 148, A1341–A1345. 10.1149/1.1415547.

[ref35] Stoerzinger K. A.; Hong W. T.; Crumlin E. J.; Bluhm H.; Shao-Horn Y. Insights into Electrochemical Reactions from Ambient Pressure Photoelectron Spectroscopy. Acc. Chem. Res. 2015, 48, 2976–2983. 10.1021/acs.accounts.5b00275. 26305627

[ref36] Kienast G.; Verma J.; Klemm W. Das Verhalten Der Alkalimetalle Zu Kupfer, Silber Und Gold. Z. Anorg. Allg. Chem. 1961, 310, 143–169. 10.1002/zaac.19613100304.

[ref37] Rehnlund D.; Valvo M.; Tai C. W.; Angstrom J.; Sahlberg M.; Edstrom K.; Nyholm L. Electrochemical Fabrication and Characterization of Cu/Cu2o Multi-Layered Micro and Nanorods in Li-Ion Batteries. Nanoscale 2015, 7, 13591–13604. 10.1039/C5NR03472H. 26206712

[ref38] Valvo M.; Rehnlund D.; Lafont U.; Hahlin M.; Edstrom K.; Nyholm L. The Impact of Size Effects on the Electrochemical Behaviour of Cu2o-Coated Cu Nanopillars for Advanced Li-Ion Microbatteries. J. Mater. Chem. A 2014, 2, 9574–9586. 10.1039/c4ta01361a.

[ref39] Riess I. What Does a Voltmeter Measure?. Solid State Ionics 1997, 95, 327–328. 10.1016/S0167-2738(96)00542-5.

[ref40] Shavorskiy A.; Ye X.; Karslıoğlu O.; Poletayev A. D.; Hartl M.; Zegkinoglou I.; Trotochaud L.; Nemšák S.; Schneider C. M.; Crumlin E. J.; Axnanda S.; Liu Z.; Ross P. N.; Chueh W.; Bluhm H. Direct Mapping of Band Positions in Doped and Undoped Hematite During Photoelectrochemical Water Splitting. J. Phys. Chem. Lett. 2017, 8, 5579–5586. 10.1021/acs.jpclett.7b02548. 29083905

[ref41] Favaro M.; Valero-Vidal C.; Eichhorn J.; Toma F. M.; Ross P. N.; Yano J.; Liu Z.; Crumlin E. J. Elucidating the Alkaline Oxygen Evolution Reaction Mechanism on Platinum. J. Mater. Chem. A 2017, 5, 11634–11643. 10.1039/C7TA00409E.

[ref42] Stoerzinger K. A.; Favaro M.; Ross P. N.; Hussain Z.; Liu Z.; Yano J.; Crumlin E. J. Stabilizing the Meniscus for Operando Characterization of Platinum During the Electrolyte-Consuming Alkaline Oxygen Evolution Reaction. Top. Catal. 2018, 61, 2152–2160. 10.1007/s11244-018-1063-6.

