# Supplementary material for: Probing Electrochemical Potential Differences over the Solid/Liquid Interface in Li-Ion Battery Model Systems
Source: ACS Appl Mater Interfaces. 2021 Jul 12;13(28):32989–96. doi: 10.1021/acsami.1c07424 (PMC8397238; doi:10.1021/acsami.1c07424)
Supplement: Supplementary file 1 — am1c07424_si_001.pdf [file am1c07424_si_001.pdf]

# Supporting information

## Probing Electrochemical Potential Differences Over the Solid/Liquid Interface in Li-ion Battery Model Systems

Ida Källquist[a], Fredrik Lindgren[a], Ming-Tao Lee[b], Andrey Shavorskiy[c], Kristina Edström[b], Håkan Rensmo[a], Leif Nyholm[b], Julia Maibach[d,*], Maria Hahlin[a,b,*]

[a] Department of Physics and Astronomy, Uppsala University, 751 20 Uppsala, Sweden
[b] Department of Chemistry - Ångström, Uppsala University, 751 20 Uppsala, Sweden
[c] MAX IV Laboratory, Lund University, 225 94 Lund, Sweden
[d] Institute for Applied Materials (IAM), Karlsruhe Institute of Technology (KIT), Hermann-von-Helmholtz-Platz 1, 76344 Eggenstein-Leopoldshafen, Germany

[*] corresponding authors
E-mail: maria.hahlin@kemi.uu.se (Maria Hahlin), julia.maibach@kit.edu (Julia Maibach)

## 1. Characterization of the Cu WE

Figure S1 shows the measured Cu 2p, Cu LMM and O 1s spectra of the pristine Cu electrode. By comparing the spectra to reference spectra of Cu and Cu oxide,[1] it can be concluded that the Cu metal is covered with a Cu oxide layer dominated by $Cu_2O$.

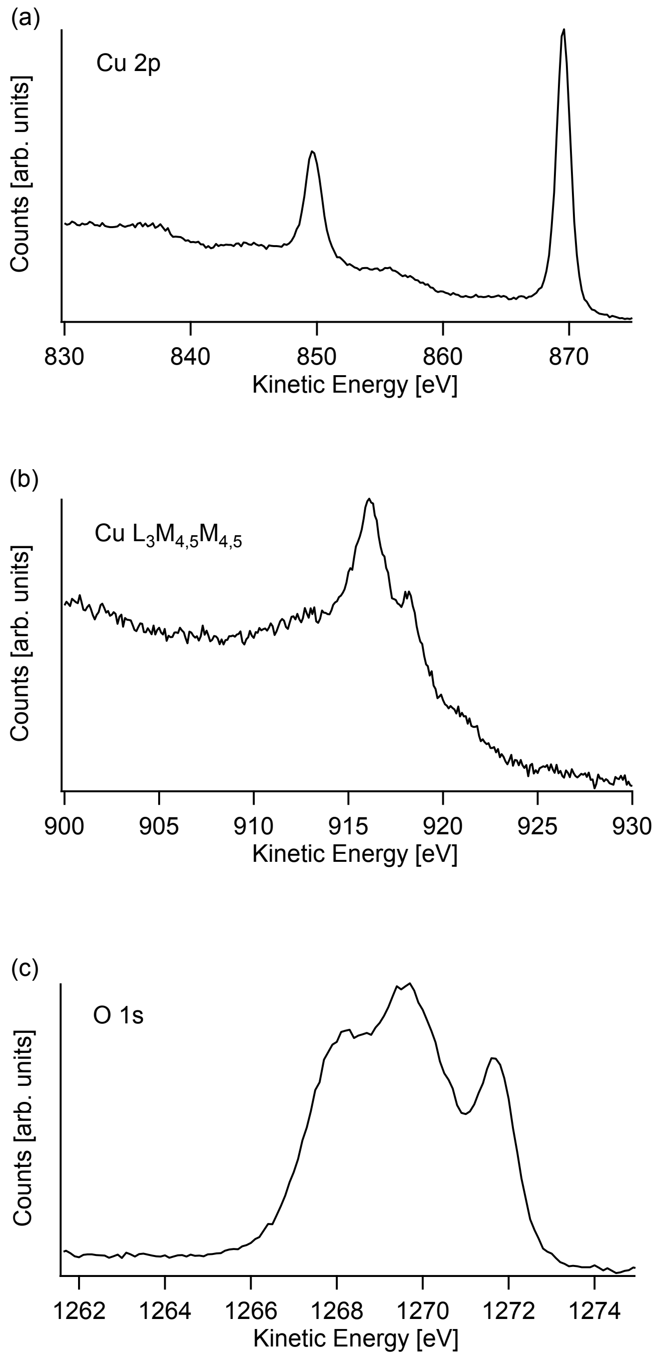


*Figure S1: Spectra of the pristine Cu-WE, (a) Cu 2p, (b) Cu L3M4,5M4,5 and (c) O 1s.*

## 2. Electrochemical measurements

Figures S2 and S3 show the first and second cycle of the Cu electrode, performed with cyclic voltammetry. The CVs show Cu reduction (starting at ~3 V with a max around 1.5 V) and SEI formation (starting around 0.8 V). Figure S4 and S5 show the electrochemical measurements performed during APPES for the Au and Cu WE, respectively. APPES measurements are performed under a constant potential step when the current has decayed to a stable value. Currents during APPES measurements are presented in Table S1 and S2.

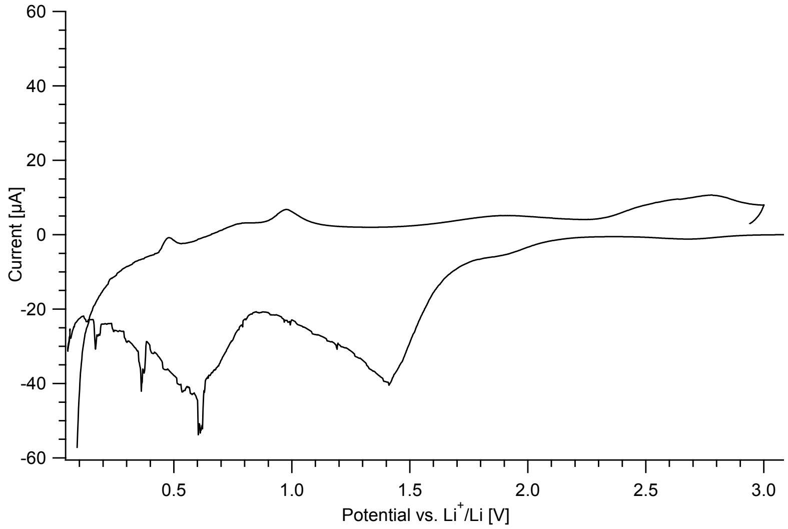


*Figure S2: CV from the first cycle of a fresh Cu-electrode measured in the same cell-setup and conditions as during APPES measurements. Scan rate was 1 mV/s, from OCV (~3.1 V) to 0.05 and back to 3.0 V. The three spikes of the cathodic current at ~0.6, ~0.4 and ~0.2 V is due to small adjustments of the sample position. The first indication of reduction of the Cu WE starts just below 3 V and the main copper oxide reduction peak is seen to start around 2 V (with the current maximum around 1.4 V). The second major peak (with a maximum around 0.6 V) is considered to originate from electrolyte reduction.*

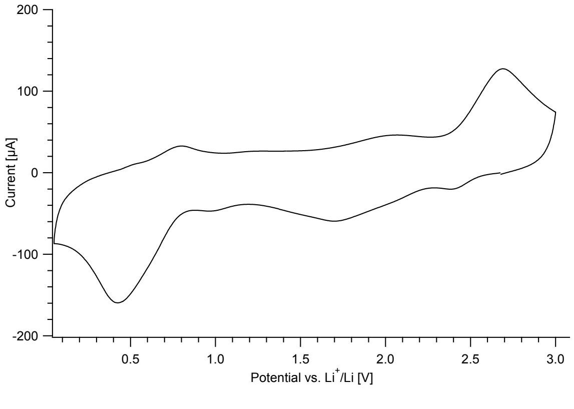


*Figure S3: CV from the second cycle of a fresh Cu-electrode measured in the same cell-setup and conditions as during APPES measurements. The scan rate is 10 mV/s, starting at ~2.7 V to 0.05 V to 3.0 V and back to ~2.7 V. During this second cycle the oxide reduction peak (now with a maximum around 1.7 V) is decreased relatively to the electrolyte reduction peak (now with a maximum at 0.4 V).*

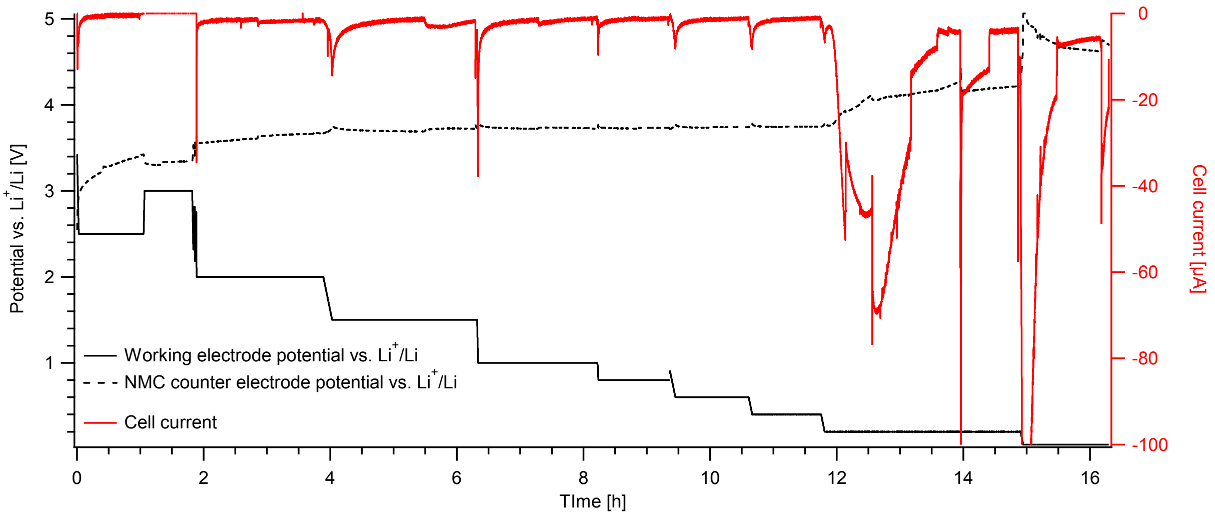


*Figure S4: Au working electrode potential, NMC counter electrode potential and cell currents during the APPES measurements.*

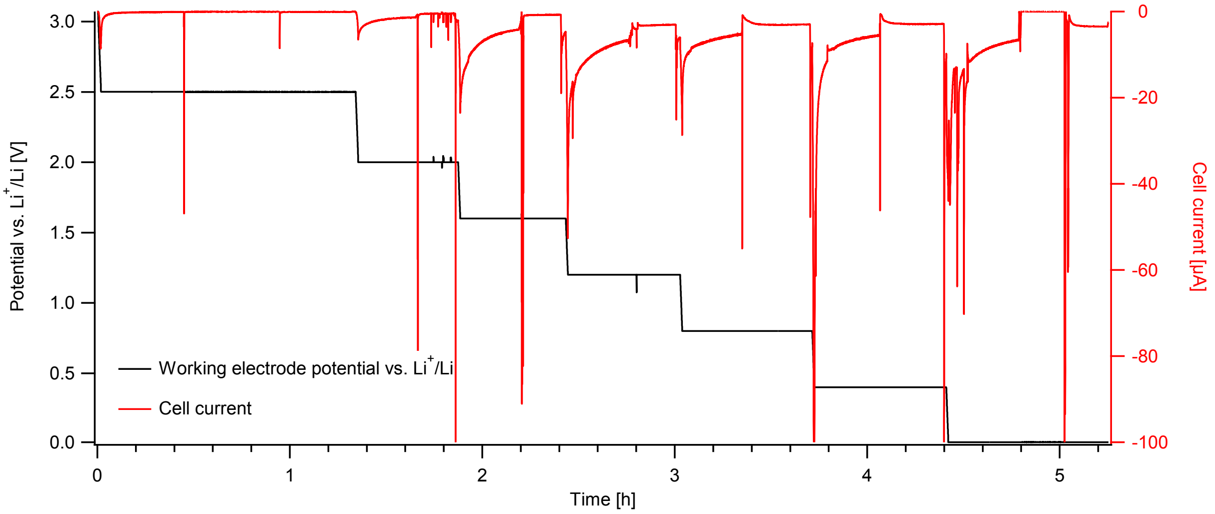


*Figure S5: Cu working electrode potential and cell currents during the APPES measurements.*

*Table S1: Currents for the Au setup during operando APPES measurements*

| Applied voltage [V | Cell current during APPES measurement (µA) |
|---|---|
| 3.0 | -0.004 |
| 2.5 | -0.5 |
| 2.0 | -2 |
| 1.5 | -3 |
| 1.0 | -2 |
| 0.8 | -2 |
| 0.6 | -2 |
| 0.4 | -2 |
| 0.2 | -20 |
| 0.05 | -7 |

*Table S2: The currents for the Cu setup during operando APPES measurements.*

| Applied voltage [V] | Cell current during APPES measurement (µA) |
|---|---|
| 2.5 | -0.5 |
| 2.0 | -0.5 |
| 1.6 | -0.8 |
| 1.2 | -3 |
| 0.8 | -3 |
| 0.4 | -3 |
| 0.01 | -3 |

### 3. Overpotentials and iR-drop in the electrolyte

If a voltage is measured during a faradaic reaction, the measured voltage vs the RE will in general not be equal to the standard/equilibrium potential of the redox reaction predicted from thermodynamics. Whenever a current is running, the measured voltage in addition contain contributions from an overpotential η, stemming from the kinetics of the chemical reaction, and from an $iR_s$-drop, stemming from the ion transport in the bulk solution. Both these contributions can be modelled as resistances in the system, but they are usually treated separately since the overpotential(s) depend on the electrochemical reaction and is attributed to the potential at the electrode (interface), while the $iR_s$-drop is independent of the interfacial chemistry and is a property of the bulk electrolyte. If there is an $iR_s$-drop in the solution, the voltage measured vs. the RE will differ from the voltage drop over the WE/electrolyte interface by the *uncompensated* $iR_s$-drop. This is illustrated in Figure S6. By choosing electrolytes with good ion conductivity and optimizing the design of the electrochemical cell, the $iR_s$-drop can be minimized.

In our setup the electrolyte conductivity (κ) for 1 M $LiClO_4$ in PC is about 10 mS $cm^{-1}$,[2] the distance between WE and CE (d) is approximately 1 cm and the WE area ($A_{WE}$) is approximately 2 $cm^2$. This means that the resistance ($R_s$) in the bulk electrolyte can be estimated.

$$R_s = \frac{\mathrm{d}}{\kappa \times A_{WE}} = \frac{1\ \mathrm{cm}}{10\ \mathrm{mS\ cm}^{-1} \times 2\ cm^2} = 50\ S^{-1} = 50\ \Omega$$

The current (i) is typically a few µA, but at most 20 µA during APPES measurements (see table S1 and S2), so the voltage drop over the electrolyte (U) can be estimated to:

$$U = R \times i = 50\ \Omega \times 20\ \mu A = 0.001\ V$$

Thus, the $iR_s$-drop in the electrolyte would be ~1 mV. Since the deviation from the expected dependence of 1 eV/V during lithiation is ~0.5 eV/V and ~0.3 eV/V for the Au and Cu WE, respectively, and the voltage change in Region B is ~0.4 V and ~0.9 V, the $iR_s$-drop is too small to account for this deviation.

It can still be noted that we certainly have mass transport limitations in the thin liquid meniscus, that slow down the rate of the reaction and give lower current densities at the same voltage due to the (additional) overpotential needed to drive the reaction. However, this will not affect $\Delta\bar{\mu}_e$ as measured by APPES on the *bulk electrolyte* since this potential drop is located at the electrode/electrolyte interface.

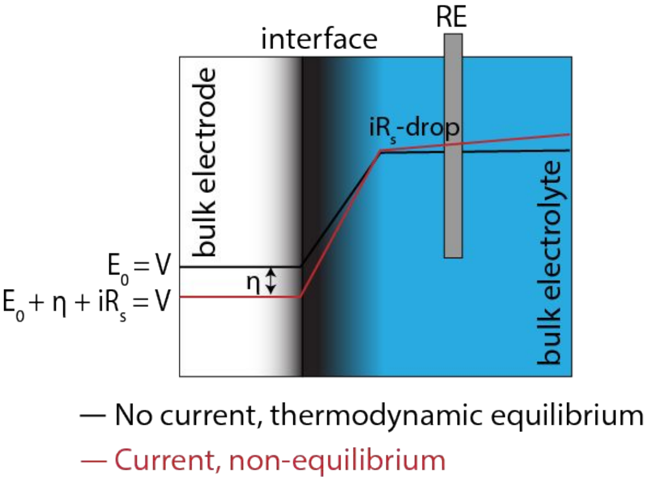


*Figure S6: Schematic illustration of the different contributions from resistances in the bulk electrolyte ($iR_s$) and from reaction kinetics at the electrode/electrolyte interface (overpotential, η).*

**Supplementary References**

1. Biesinger, M. C.; Payne, B. P.; Grosvenor, A. P.; Lau, L. W. M.; Gerson, A. R.; Smart, R. S. C., Resolving Surface Chemical States in Xps Analysis of First Row Transition Metals, Oxides and Hydroxides: Cr, Mn, Fe, Co and Ni. *Applied Surface Science* **2011**, *257*, 2717-2730.
2. Chen, H.; Fergus, J.; Jang, B., The Effect of Ethylene Carbonate and Salt Concentration on the Conductivity of Propylene Carbonate| Lithium Perchlorate Electrolytes. *J Electrochem Soc* **2000**, *147*, 399.
